# IGF-1 increases invasive potential of MCF 7 breast cancer cells and induces activation of latent TGF-β1 resulting in epithelial to mesenchymal transition

**DOI:** 10.1186/1478-811X-9-10

**Published:** 2011-05-02

**Authors:** Logan A Walsh, Sashko Damjanovski

**Affiliations:** 1 Department of Biology, University of Western Ontario, London, Ontario, N6A 5B7, Canada

## Abstract

**Introduction:**

TGF-β signaling has been extensively studied in many developmental contexts, amongst which is its ability to induce epithelial to mesenchymal transitions (EMT). EMTs play crucial roles during embryonic development and have also come under intense scrutiny as a mechanism through which breast cancers progress to become metastatic. Interestingly, while the molecular hallmarks of EMT progression (loss of cell adhesion, nuclear localization of β-catenin) are straightforward, the cellular signaling cascades that result in an EMT are numerous and diverse. Furthermore, most studies describing the biological effects of TGF-β have been performed using high concentrations of active, soluble TGF-β, despite the fact that TGF-β is produced and secreted as a latent complex.

**Methods:**

MCF-7 breast cancer cells treated with recombinant IGF-1 were assayed for metalloproteinase activity and invasiveness through a matrigel coated transwell invasion chamber. IGF-1 treatments were then followed by the addition of latent-TGF-β1 to determine if elevated levels of IGF-1 together with latent-TGF-β1 could cause EMT.

**Results:**

Results showed that IGF-1 - a molecule known to be elevated in breast cancer is a regulator of matrix metalloproteinase activity (MMP) and the invasive potential of MCF-7 breast cancer cells. The effects of IGF-1 appear to be mediated through signals transduced via the PI3K and MAPK pathways. In addition, increased IGF-1, together with latent TGF-β1 and active MMPs result in EMT.

**Conclusions:**

Taken together our data suggest a novel a link between IGF-1 levels, MMP activity, TGF-β signaling, and EMT in breast cancer cells.

## Introduction

Breast cancers, and indeed most cancers, usurp cellular signaling pathways, particularly developmental pathways, that are normally tightly regulated in the animal or embryo. Alteration of these pathways often results in oncogenic transformations. For example, in mammary epithelial cells, a malignant phenotype is characterized by increased proliferation, resistance to apoptosis and metastasis. The transforming growth factor beta (TGF-β) family of signaling molecules are key regulators of both developmental, and malignant processes. Similarly, insulin-like growth factor (IGF) signaling is also a hallmark for development and subsequent tissue homeostasis [1]. IGF signaling is comprised of a dynamic network of proteins including ligands (IGF-I and IGF-II), their associated receptors, IGF-binding proteins (IGFBPs), and IGFBP proteases [2,3]. Of particular interest, IGF-1 protein has been most strongly implicated in breast cancer progression because of its mitogenic and anti-apoptotic effect on mammary epithelial cells [4]. IGF-I can act in an endocrine, paracrine or autocrine manner. Accordingly, IGF-1 levels and activity have been closely examined in proliferative tissues for their relationship to changes in cellular morphology associated with cancer progression. Indeed there is a strong positive association between IGF-1 levels and breast cancer, especially among premenopausal women [4].

Another potent signaling molecule that is correlated with changes in cellular morphology and migration is TGF-β. TGF-β is a secreted protein that exists in three isoforms: TGF-β1, TGF--β2 and TGF-β3. Together these proteins control many cellular processes including cellular proliferation, differentiation, and altered TGF-β signaling has a significant role in a variety of diseases [5]. TGF-β signaling has been extensively studied in cancer for its ability to induce epithelial to mesenchymal transitions (EMTs) [6]. Amongst the common denominators for an EMT are reduced cell adhesion, increased cell migration and the nuclear localization of β-catenin [7,8]. Though EMTs are primarily a mechanism for tissue patterning during development, this cellular process has also been observed in adult tissue as an explanation for the conversion of various epithelial cells to a mesenchymal, and ultimately metastatic phenotype. While many studies have investigated TGF-β's role in regulating EMTs, most studies describing biological effects of TGF-β have been carried out *in vitro *using high concentrations of active, soluble TGF-β, despite the fact that TGF-β is produced and secreted *in vivo *as a latent complex [9]. While much is known about TGF-β signaling in comparison, the mechanism of TGF-β activation, and its relationship to IGF-1 in breast cancer metastasis, is poorly understood.

IGF family molecules bind to a variety of insulin-like growth factor receptors (IGFRs). These receptors are receptor tyrosine kinases (RTKs) and most frequently signal through PI3K and MAPK dependent mechanisms [10]. Conversely, TGF-β receptors are serine/threonine kinases that typically signal through SMAD 2/3 dependent mechanisms [11]. Of particular interest, TGF-β is secreted as a preproprotein and is part of an inactive complex. This complex consists of the TGF-β, a propeptide latency-associated peptide (LAP), and a latent TGF-β binding protein (LTBP). Once secreted this complex can then be linked to components of the extracellular matrix (ECM). Typically the majority of extracellular TGF-β is inactive. Only upon the activation of this latent complex is a mature active dimeric TGF-β peptide released. Several means of TGF-β activation have been elucidated, but all involve the proteolytic degradation of LAP, with plasmin and MMPs proving to be important players in the degradation of LAP and the release of mature TGF-β. Interestingly, however, binding of LAP to integrins or other ECM proteins such as thrombospondin, can cause a conformational change in LAP and also result in the subsequent release of active TGF-β, which is then free to bind to its receptor [12,13].

In this study we look at the effects of both IGF-1 stimulation, and increased latent TGF-β1 levels, on MCF-7 breast cancer cells to see if these proteins may work in conjunction to alter cellular morphology and increase the invasive potential of these cells. Here we demonstrate a novel link between IGF-1 signaling, MMP activation and TGF-β signaling, and propose a novel mechanism for EMT induction in MCF-7 cells.

## Results

### IGF-1 regulated metalloproteinase activity in MCF-7 breast cancer cells via the PI3K and MAPK pathways

**Figure 1:**
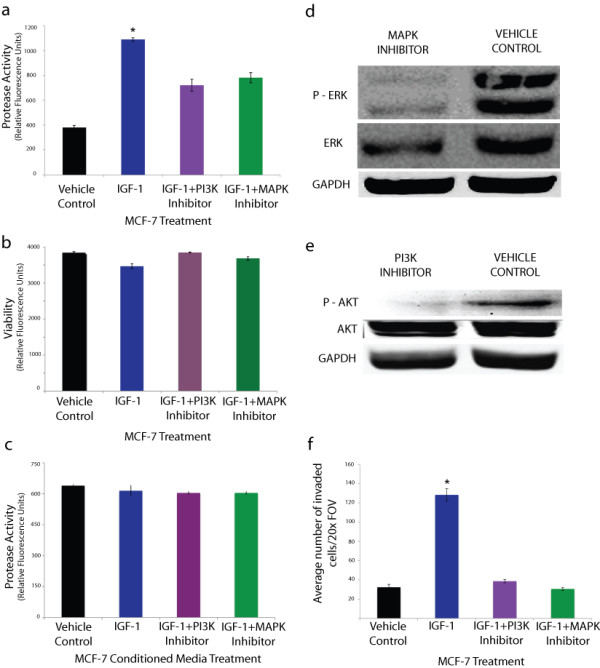
**IGF-1 increased metalloproteinase activity and invasiveness in MCF-7 breast cancer cells via the PI3K and MAPK pathways**. a) Treatment of MCF-7 cells with 100 nM recombinant IGF-1 caused a 2.9 fold increase in protease activity as determined using a fluoregenic metalloproteinase substrate. Pretreatment of cells with PI3K or MAPK inhibitors attenuated the increased protease activation and resulted in a 34% and 29% decrease in activity respectively compared to IGF-1 treatment alone. b) MCF-7 cell viability, assessed by a fluorogenic viability assay, was not significantly affected by IGF-1 and/or inhibitor treatments. c) Treatment of MCF-7 conditioned media with aforementioned reagents resulted in no significant changes in MMP activity. d-e) Western Blot analysis of MCF-7 cells treated with MAPK or PI3K inhibitors reveals decreased expression of phospho-ERK and phospho-AKT expression respectively. f) Treatment of MCF-7 cells with 100 nM recombinant IGF-1 increased invasiveness through a matrigel coated transwell chamber by ~400% compared to vehicle treatment (control). Pre-treatment of cells with PI3K or MAPK inhibitors before IGF-1 treatment resulted in no significant change in invasiveness compared to vehicle control. Each assay was repeated three times (three experimental repeats). All data are mean ± s.e.m, (*n *= 3) *P < 0.01.

IGF-1 has been shown previously to stimulate protease activity in many cell types [13]. To determine if IGF-1 could stimulate metalloproteinase activity in MCF-7 cells they were treated with 100 nM recombinant IGF-1, and subsequently their conditioned media was used to assay for metalloproteinase activity using a broad-spectrum metalloproteinase (Mca-KPLGL-Dpa-AR-NH2) Fluorogenic Peptide Substrate. IGF-1 treatment resulted in a 2.9 fold increase in metalloproteinase activity compared to vehicle control (Figure 1a). To identify the pathway(s) regulating protease induction by IGF-I, pharmacological inhibitors were used that inhibit Phosphoinositide 3-kinases (PI3K) and Mitogen-Activated Protein Kinase (MAPK) signaling - two known downstream IGF-I effectors [13]. Treatment with the PI3K inhibitor wortmannin (5 nm) significantly reduced IGF-I-mediated protease activation by 34% (Figure 1a). Treatment with the MAPK inhibitor SB 202190 (10 M) had a 29% decrease in IGF-1 induced protease activation (Figure 1a). Cell viability was unaltered by treatments (Figure 1b) confirming that the effects of the inhibitors were not due to alterations in cell viability. The specificity of the reagents on mediating cellular events and not simply altering activity in the culture media was assayed via the addition of the above reagents to cell free conditioned media where in resulted in no significant changes in MMP activity (Figure 1c). Western Blot analysis of MCF-7 cells treated with MAPK or PI3K inhibitors revealed decreased levels of phospho-ERK and phospho-AKT moieties respectively, confirming the efficacy of the inhibitors (Figure 1d-e). Notably, the treatment of MCF-7 cells with IGF-1 increased invasiveness through a matrigel coated transwell invasion chamber by ~400% compared to control (Figure 1f). Pre-treatment of cells with PI3K or MAPK inhibitors before IGF-1 treatment resulted in no significant change in invasiveness compared to control (Figure 1f).

### IGF-1 with latent TGF-β1 caused morphological changes in MCF-7 cells consistent with EMT

**Figure 2:**
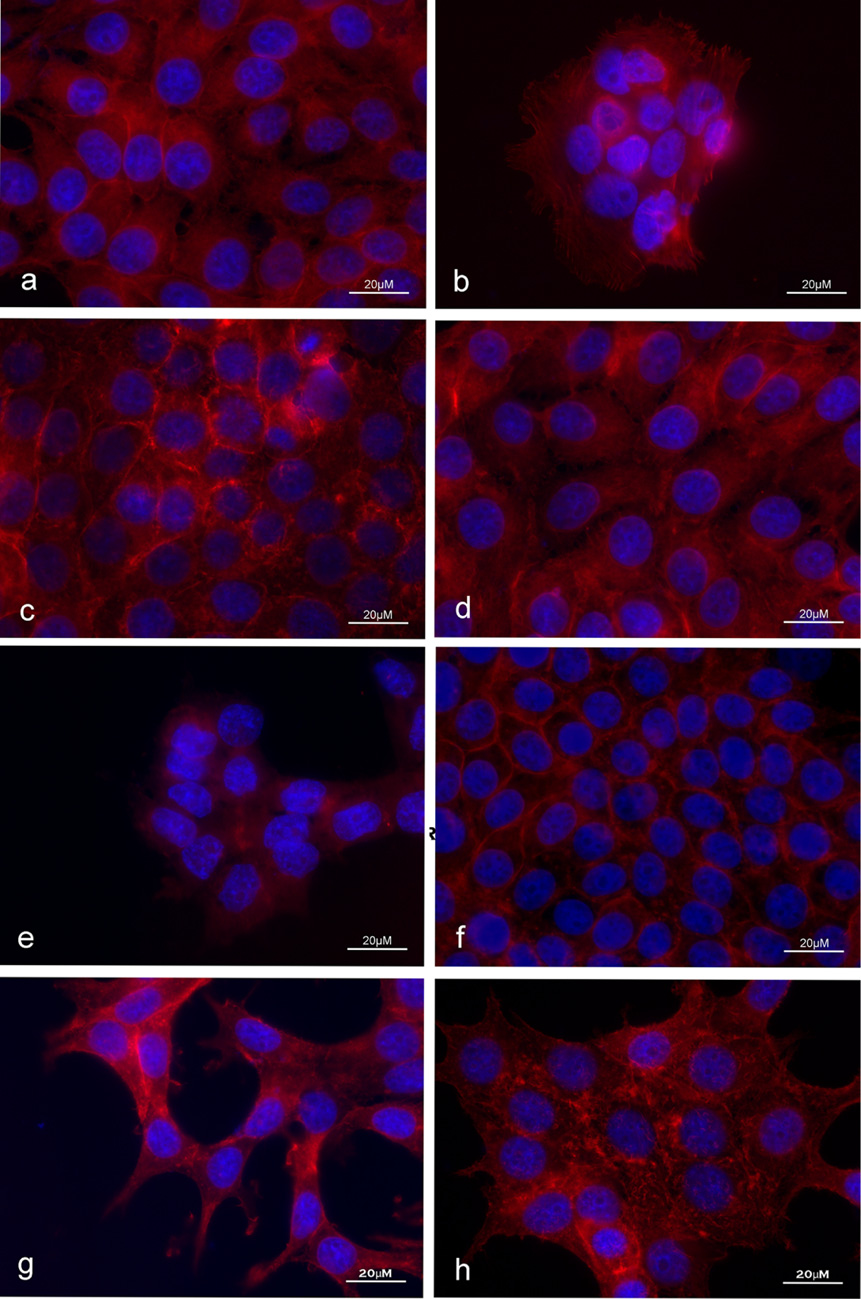
**IGF-1 and latent TGF-β1 caused morphological changes in MCF-7 cells consistent with EMT**. Vehicle treated cells showed typical rounded epithelial morphology (a). Treatment of MCF-7 cells with 100 nM recombinant IGF-1 + 10 nM latent TGF-β1 caused a mesenchymal morphological phenotype characterized by a fibroblast-like appearance (g). Treatment with IGF-1 or latent TGF-β1 alone did not result in a morphological change (c, e). Pre-treatment of cells with PI3K or MAPK inhibitors abolished IGF-1 + latent TGF-β1 induced mesenchymal morphological phenotype (d, f). BB94 or TGF-β1 inhibitor prevented the morphological changes associated with IGF-1 + TGF-β1 treatment (b, h). Cytoskeleton was stained with phalloidin (red) and the nucleus with DAPI (blue). Images are representative of 3 independent experiments with consistent results.

Recent evidence suggested that signaling after the proteolytic activation of latent TGF-β1 could induce EMT in breast cancer leading to metastasis [14]. There is also a strong correlation between high levels of IGF-1 signaling and metastasis [15]. The link between IGF-1 and TGFβ1 signaling however, has not thoroughly been explored. Here we show that treating MCF-7 cells first with IGF-1 (8 h), and then subsequently with latent TGF-β1 (48 h) resulted in a change to a cellular fibroblast-like morphology consistent with EMT (Figure 2g). MCF-7 vehicle treatment (control), or treatment with IGF-1 or latent TGF-β1 alone did not induce this change in cellular morphology (Figure 2a, c, e). Furthermore, pretreatment of cells with PI3K or MAPK inhibitors demonstrated the specificity of these pathways in IGF-1 mediated latent TGF-β1 induced morphological changes as it prevented changes in morphology (Figure 2d, f). TGF-β1 specificity is highlighted by the addition of TGF-β inhibitor that blocked changes in morphology after IGF-1+latent TGF-β1 treatment (Figure 2h). Inhibition of matrix metalloproteinase's using BB94 also abolished IGF-1 mediated, latent TGF-β1 signals, as cells retained epithelial morphology (Figure 2b). These findings indicated that IGF-1 treatment may have played a role in promoting the activation of latent TGF-β1 leading to EMT.

### IGF-1 with latent TGF-β1 resulted in changes in marker gene expression consistent with EMT

**Figure 3:**
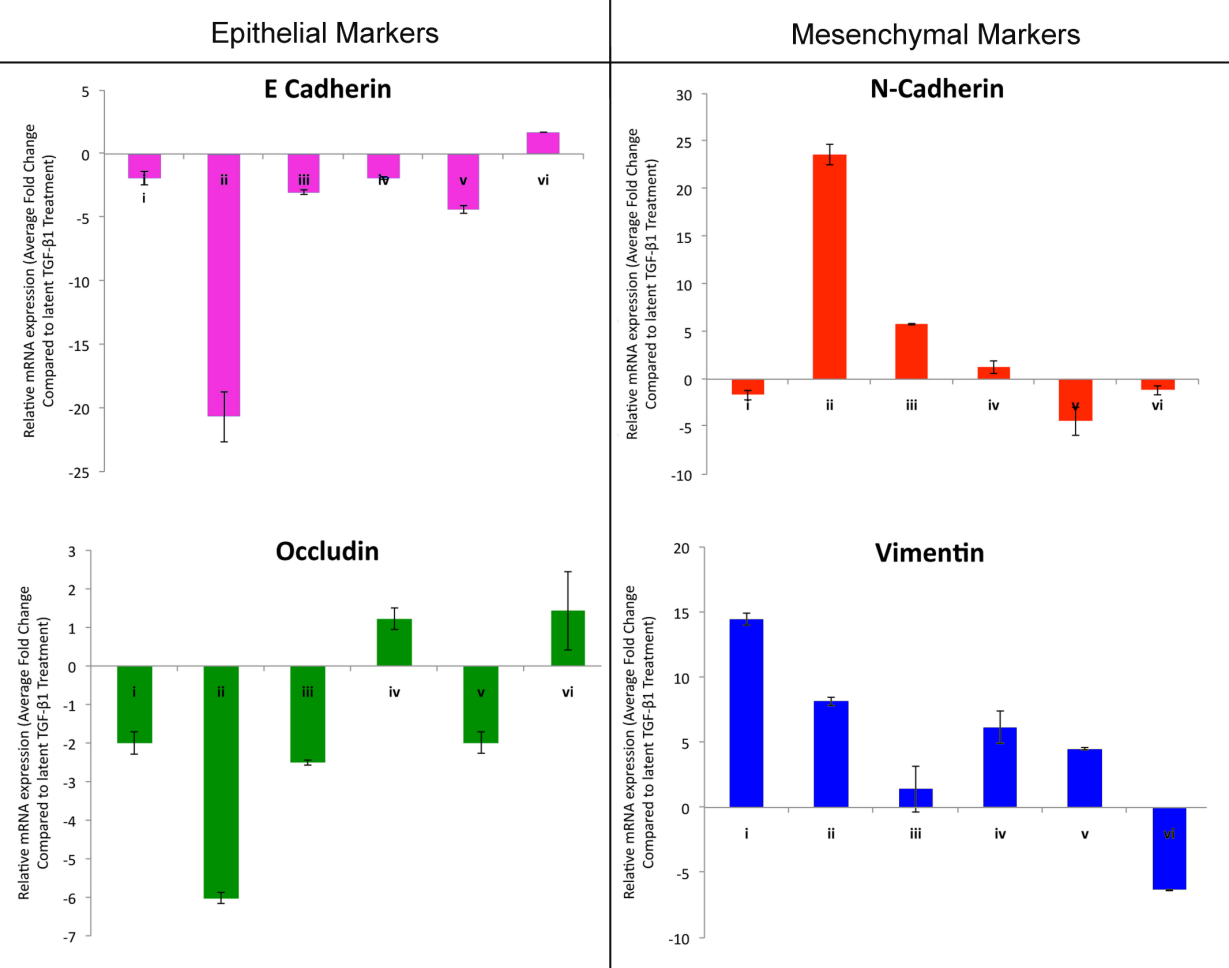
**Semi-quantitative real time PCR showed IGF-1 and latent TGF-β1 resulted in changes in marker gene expression consistent with EMT in MCF-7 cells**. MCF-7 cells treated with (i) IGF-1, (ii) IGF-1+latent TGF-β1, (iii) IGF-1+latent TGF-β1+PI3K inhibitor, (iv) IGF-1+latent TGF-β1+MAPK inhibitor, (v) IGF-1atent TGF-β1+TGF-β1 inhibitor or (vi) IGF-1+latent TGF-β1+MMP inhibitor were analyzed for levels of marker genes associated with EMT. E-cadherin, and occludin levels decreased by 21 and 6 fold respectively while N-cadherin and vimentin levels increased by 24 and 8 fold respectively when treated with IGF1-+ latent TGF-β1 as compared to latent TGF-β1 treatment alone. As there was no significant difference between vehicle and latent TGF-β1 treatment, latent TGF-β1 treatment alone was used for comparison with other treatments to further highlight changes associates with the activation of TGF-β1 and EMT. The addition of PI3K, MAPK, MMP or TGF-β1 inhibitors attenuated these changes in marker gene expression. With the exception of vimentin, IGF-1 treatment alone caused changes in marker gene expression similar to that seen with IGF-1+ TGF-β1+inhibitors. All data are mean ± s.e.m. (n = 3, three experimental repeats).

To investigate if the IGF-1 and latent TGF-β1 induced changes in morphology were consistent with changes in expression of EMT marker genes, real-time PCR was performed with primers for E-cadherin, N-cadherin, occludin and vimentin. Treatment with latent TGF-β1 alone did not promote significant changes in marker gene expression compared to vehicle treatment (control -data not shown). Expression levels after latent TGF-β1 treatments were therefore used for comparisons with other treatments. MCF-7 cells treated with IGF-1 + latent TGF-β1 showed ~24 and ~8 fold increases in mesenchymal markers N-cadherin and vimentin respectively, and ~21 and ~6 fold decreases in epithelial markers E-cadherin and occludin respectively compared to cells treated with latent TGF-β1 alone (Figure 3). This expression pattern is consistent with EMT seen in other cell types [16]. Pretreatment of cells with PI3K or MAPK inhibitors attenuated the IGF-1 + latent TGF-β1 mediated changes in EMT marker gene expression (Figure 3). IGF-1 treatment alone increased vimentin, but this in itself is not indicative of EMT. MCF-7 cells treated with a TGF-β1 inhibitor or the MMP inhibitor BB94 also did not show changes in marker gene expression consistent with EMT.

**Figure 4:**
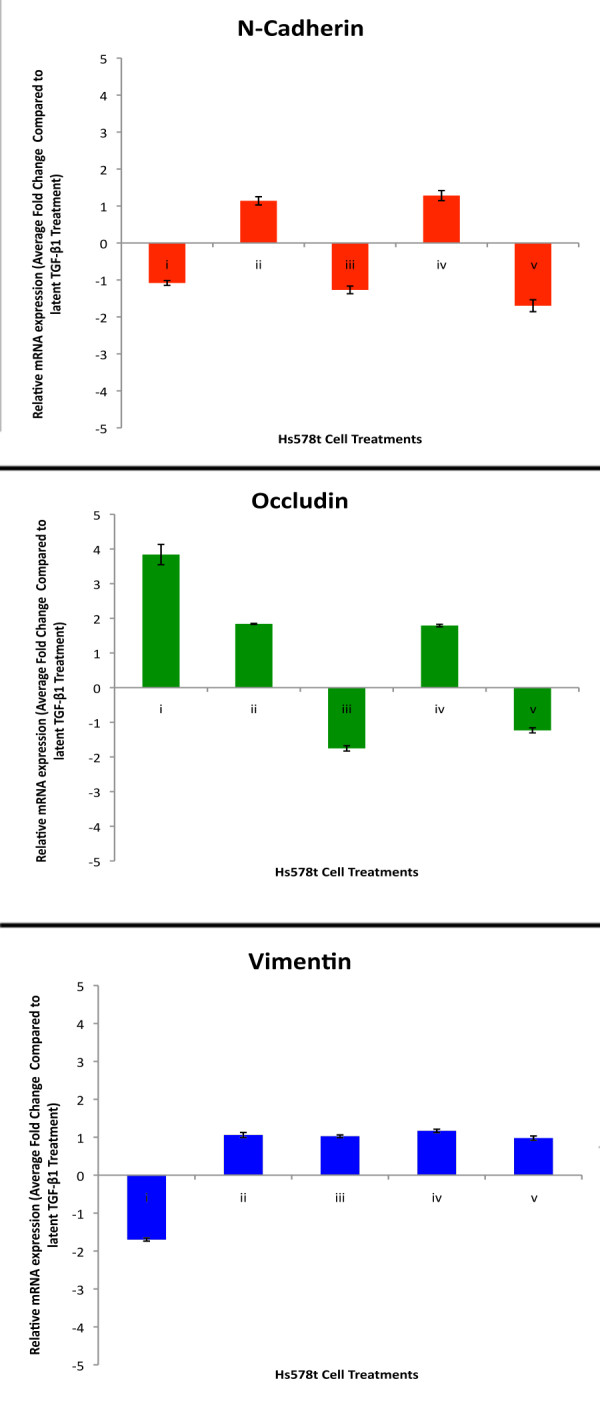
**Semi-quantitative real time PCR showed IGF-1 and latent TGF-β1 resulted in no changes in EMT marker gene expression consistent with EMT in Hs578t cells**. Hs578t cells treated with (i) IGF-1, (ii) IGF-1+latent TGF-β1, (iii) IGF-1+latent TGF-β1+PI3K inhibitor, (iv) IGF-1+latent TGF-β1+MAPK inhibitor or (v) IGF-1+latent TGF-β1+TGF-β1 inhibitor were analyzed for levels of marker genes associated with EMT. Changes in N-cadherin, occludin, and vimentin expression were not consistent with EMT after treatment with IGF-1+TGF-β1 compared to TGF-β1 treatment alone. The addition of PI3K. MAPK or TGF-β1 inhibitors did not affect these changes in marker gene expression. Hs578t cells treated with TGF-β1 alone resulted in changes in marker gene expression that were also not consistent with EMT compared to vehicle control. All data are mean ± s.e.m. (n = 3, three experimental repeats).

To investigate the specificity of the IGF-1/latent TGF-β treatments in modulating EMT specific cellular events, a cell line that could not undergo an EMT was also used. In contrast to MCF-7 cells, Hs578t breast cancer cells are highly invasive atypical epithelial cells, do not express E-cadherin, and already have a mesenchymal morphology. Although treatment of Hs578t cells with IGF-1 and/or latent TGF-β1 resulted in minor alterations of EMT marker gene expression, there were no significant changes in multiple marker genes that would be consistent with, and indicative of an EMT signature (Figure 4) [17].

### IGF-1 with latent TGF-β1 resulted in nuclear localization of β-catenin in MCF-7 cells

**Figure 5:**
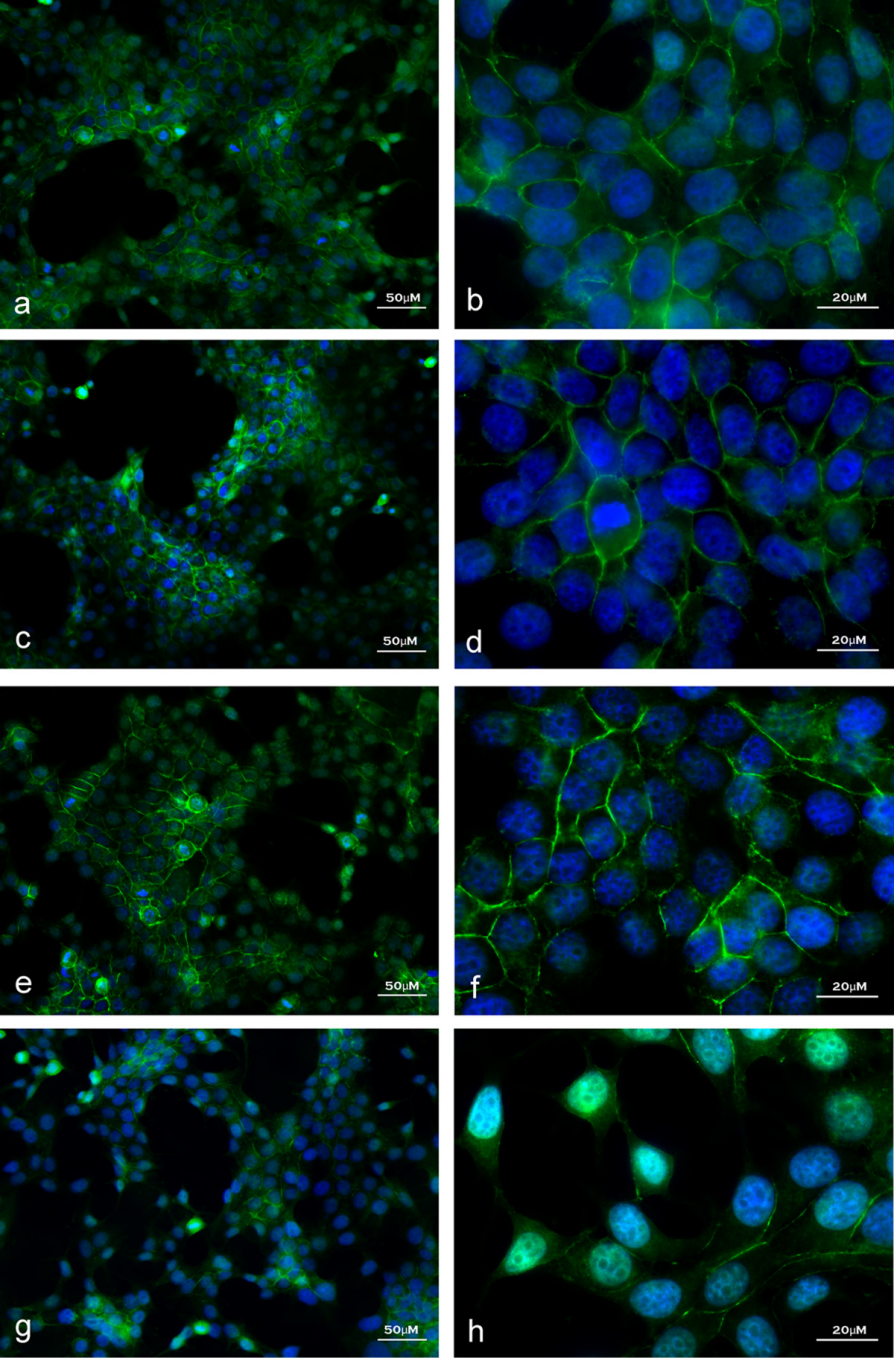
**IGF-1 and latent TGF-β1 resulted in nuclear localization of β-catenin in MCF-7 cells**. Nuclei are stained with DAPI (blue) while β-catenin is localized with a specific antibody (green). Treatment of MCF-7 cells with 100 nM recombinant IGF-1 + 10 nM TGF-β1 resulted in nuclear (blue) localization of β-catenin (green) (green blue overlap g-h). Treatment with vehicle control (a-b), IGF-1 (c-d) or TGF-β1 alone (e-f) showed β-catenin primarily localized at the plasma membrane. Images are representative of 3 independent experiments with consistent results.

There have been many contradictory studies regarding the induction of the β-catenin/TCF pathway by IGF [18]. Playford et al., reported that IGF alone could not induce β-catenin/TCF-dependent transcriptional activation whereas other researchers have reported it can. Since activation of this pathway and the subsequent nuclear localization of β-catenin is a hallmark of EMT, we chose to look at the nuclear localization of β-catenin after IGF-1 and latent TGF-β1 treatment of MCF-7 breast cancer cells. IGF-1 + latent TGF-β1 treatment of MCF-7 cells resulted in nuclear localization of β-catenin (Figure 5g, h). Vehicle (control), IGF-1 or TGF-β1 alone did not cause nuclear localization of β-catenin as the β-catenin signal was still prevalent at the plasma membrane (Figure 5a-f).

## Discussion

The extensive array of cytokines and growth factors sequestered by the ECM can regulate cell proliferation, differentiation and migration [19]. Accordingly, the importance of maintaining homeostasis within the ECM microenvironment has been widely recognized and the significance of ECM remodeling has recently become the focus of intense scientific research. The diversity of the ECM underlies its function as a structural element and as a contributor to tissue development and physiology [20]. MMPs are a family of highly homologous endopeptidases that cleave and remodel the extracellular matrix (ECM) [21]. Currently, there are 24 known MMPs that collectively have the potential to cleave and remodel all major ECM constituents. Considering the range of effects that MMPs and ECM remodeling can have on cell physiology, it is not surprising that MMPs activity has been associated with many diseases and pathological conditions including the metastatic potential of many types of cancer [22].

Here we demonstrate that IGF-1 plays a role in the activation of MMPs and causes increased invasive potential in breast cancer cells. Specifically we show that IGF-1 increases the proteolytic activity and invasive potential of MCF-7 cells. Mca-KPLGL-Dpa-AR-NH2 is a fluorogenic peptide substrate that is a highly specific target for many MMPs including: MMP-1 (collagenase 1), MMP-2 (gelatinase A), MMP-7 (matrilysin), and MMP-9 (gelatinase B). The use of this peptide demonstrated that the addition of IGF-1 alone increased MMP activity.

Inhibition of either the PI3K or MAPK pathways illustrated the specificity of this IGF-1 mediated increase in MMP activity. It has been previously shown that both PI3K and MAPK pathways are activated by IGF-1 to promote cell survival, but to our knowledge, it has never been shown that IGF-1 activates MMPs in a breast cancer cell line and that this, in part, functions via the PI3K and MAPK pathways.

Although IGF-1 signaling in breast cancer has been primarily associated with paracrine activity, endocrine sources of IGF-1 may also have a role in breast cancer. Circulating IGF-1 levels are higher in breast cancer patients compared with normal controls [23]. Breast cancer cells, as with most embryonic cells, are known to respond to many stimuli, including growth factors and cytokines that are housed in the ECM. The release of such factors could thus trigger the cells to proliferate or change their morphology, and in the case of breast cancers, metastasize. As a principal feature of breast cancer is uncontrolled proliferation, identifying and understanding the regulatory molecules and pathways that govern these cells is essential. Here we show that IGF-1 activates matrix metalloproteinase that have the potential to cleave the ECM and may release potent bioactive molecules leading to possible growth, proliferation and metastasis of a primary tumor. This reiterates the importance of IGF-1 mediated protease activation, and the downstream consequences of increased IGF-1 signaling in cell migration, invasion, and breast cancer.

**Figure 6:**
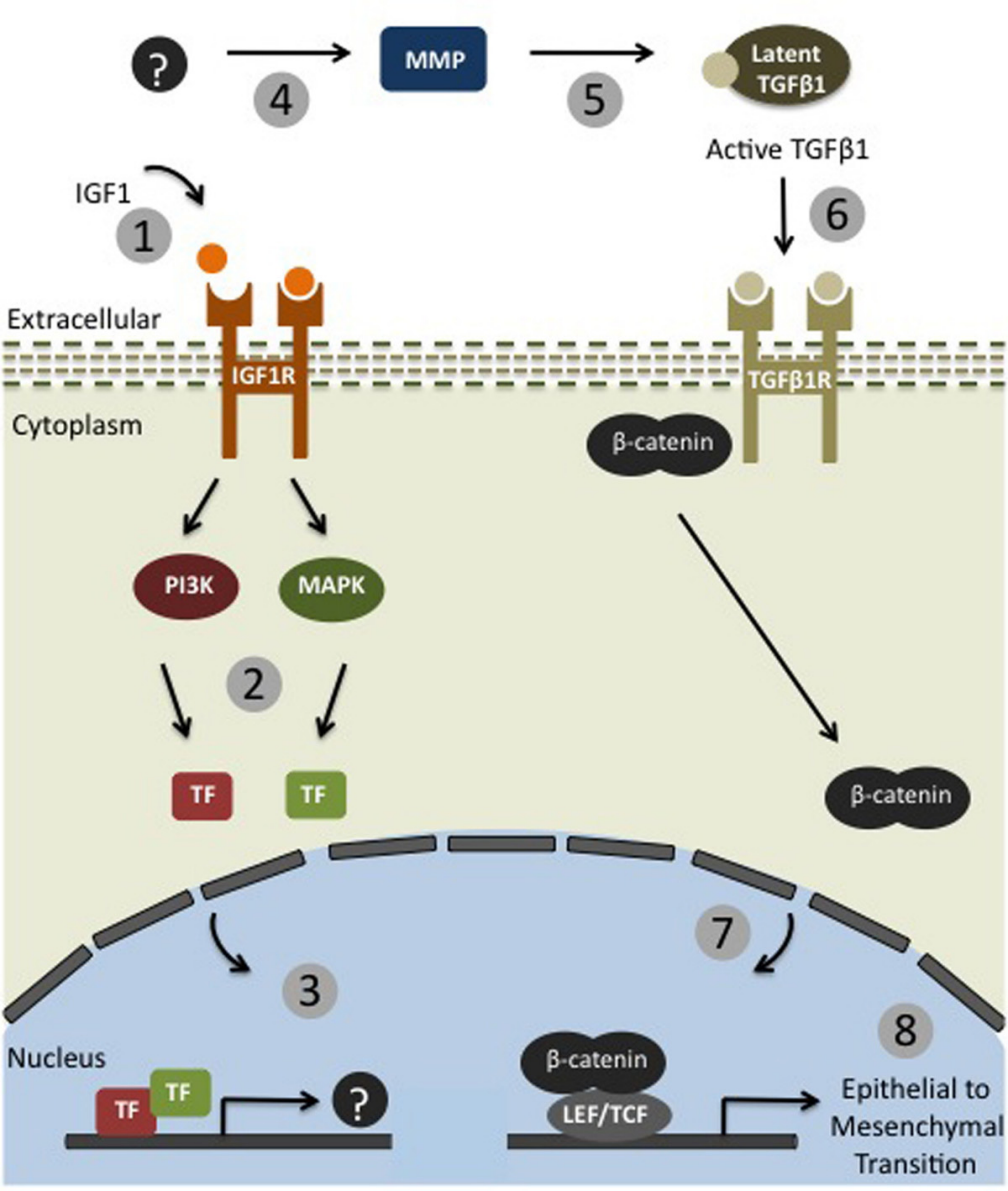
**Schematic Model of IGF-1 and TGF-β1 signaling in MCF-7 breast cancer cells**. 1) IGF-1 binds IGF-1 receptor. 2) IGF-1 receptor signals through the PI3K and MAPK pathways. 3) IGF-1 signaling causes transcriptional activation of unknown target genes (?). 4) Unknown target protein(s) (?) are secreted and activate MMPs. 5) Active MMPs are available to activate latent molecules in the ECM, such as TGF-β. 6) Active TGF-β1 binds TGF-β1 receptor. 7) TGF-β signaling causes nuclear localization of β-catenin. 8) TGF-β signaling causing transcriptional activation of genes that result in an EMT.

A major conclusion from our study is that IGF-1 signaling and TGF-β signaling work in concert to bring about an epithelial to mesenchymal transition in MCF-7 cells. TGF-β signaling has been widely studied in cancer for its ability to induce EMTs leading to metastasis [24]. Most studies describing the biological effects of TGF-β have been carried out *in vitro *using high concentrations of active, soluble TGF-β, despite the fact that TGF-β is produced and secreted *in *vivo as a latent complex [8]. Thus the conditions for the activation of TGF-β are not addressed. In addition, although both IGF-1 levels and TGF-β signaling are associated with the metastatic potential of breast cancer, the mechanism of TGF-β activation and its effect on cell invasion is still poorly understood. Here for the first time we establish a critical link between these two pathways and their contribution to cancer progression. Based on our findings in MCF-7 cells, we present a model of putative latent TGF-β1 activation (Figure 6). We propose that IGF-1 transmits signals via both the PI3K and MAPK pathways that cause the transcription of unknown but specific genes. These gene products result in the extracellular activation of MMPs. This MMP activation is IGF-1 dependent and attenuated if the PI3K or MAPK signals are blocked. MMPs are then capable of working in the ECM to activate latent TGF-β1. Active TGF-β1 results in EMT characterized by the nuclear localization of β-catenin, changes in morphology and the transcription of EMT marker genes. The addition of a TGF-β inhibitor blocks this signaling cascade, demonstrating the specificity of TGF-β1 signaling. While our finding are based on studies using MCF-7 cells, the pathways and molecules examined are globally used in many cell types, developmental processes and diseases, and as such we believe that our model has broad relevance.

Latent TGF-β activation has been described as a complex multistep process[21,25]. TGF-β maturation is highly controlled in order to regulate the formation of active TGF-β. Although there has been significant progress in elucidating the components and mode of TGF-β secretion, little is known about the fate of latent TGF-β after its association with the ECM, nor the final steps of TGF-β activation. Various evidence for latent TGF-β activation suggests its cleavage, and subsequent activation, is mediated by plasmin. The formation of active TGF-β has been shown to be blocked by plasmin inhibitors or by depletion of plasminogen [7]. Here we show a novel mechanism for TGF-β signaling via IGF-1 mediated activation of MMPs, as latent TGF-β1 activity is blocked by an MMP inhibitor. Interestingly, there is a strong positive correlation between MMP activity and the levels of plasmin, as plasmin is a post-translational activator of MMPs [25]. Perhaps in previous studies the depletion of plasmin also may have resulted in decreased activation of MMPs, which in turn may have contributed to the decreased activation of latent TGF-β (a hypothesis that we will test at a later date).

## Conclusions

Here we demonstrate a novel link between 2 hallmark signaling pathways associated with the migratory and invasive potential of cells. IGF-1 signaling ultimately induces the activation of latent TGF-β1 leading to EMT in a breast cancer cell line. Furthermore, consistent with IGF-1 mediated invasiveness, inhibition of the PI3K or MAPK pathway attenuates this IGF-1 mediated TGF-β1 signaling. This study gives further insight into the specific pathways that are required for latent TGF-β activation and highlights the importance of IGF-1 levels and TGF-β activity in cells, particularly breast cancer cells. Further experiments are required to elucidate the additional elements and the complete mechanism for IGF-1 signaling and TGF-β1 induction pathways and to confirm that these mechanisms are pertinent in other cell lines, disease and developmental pathways.

## Materials and methods

### Cell culture and treatments

The MCF-7 and Hs578t cell lines were obtained from the American Type Culture Collection (Manassas, VA) and cultured in Dulbecco's modified eagle's medium (DMEM-F12; Invitrogen Canada Inc.) supplemented with 10% fetal bovine serum (FBS; Invitrogen Canada Inc.) 50 g/ml penicillin and 50 g/ml streptomycin (Invitrogen Canada Inc.). All cells serum starved in DMEM-F12 for 24 h prior to treatments. IGF-1 (R&D Systems Inc.) was used to treat cells at 100 nM. Latent TGF-β1 (R&D Systems Inc.) was used at 10 nM. Inhibitors used were; PI3K inhibitor Wortmannin -100 nM, p38/MAPK inhibitor SB 202190 - 25 M, TGFβR1 kinase inhibitor SD 208 - 1 M (Sigma-Aldrich), MMP inhibitor BB94 - 60 nM (Tocris Biosciences). MCF-7 cells were pretreated with pharmacological inhibitors for 30 minutes prior to the addition of IGF-1. Latent TGF-β1 was added 8 h (to allow sufficient time for products of IGF-1 signaling to be translated) after the addition of IGF-1. Cells were then incubated for 48 hours prior to β-catenin localization, viability, PCR, and morphology assays. Vehicle treatments were a combination of 1× Phosphate Buffered Saline (IGF-1 vehicle) and dimethyl sulfoxide (pharmological inhibitor vehicle). Viability of cells was assessed using a DHL™ Cell Viability and Proliferation Assay Kit (Anaspec) as per manufacturer's instructions. Cell viability was quantified by measuring fluorescence with an excitation at 590 nm and emission at 530 nm using a Molecular Devices M2E microplate reader.

### MMP Activity Assay

MCF-7 cells were treated with vehicle (control), IGF-1 or IGF-1 after pretreatment with MAPK or PI3K inhibitors for 30 mins. Eight hours after the addition of IGF-1, MMP activity was assayed in the conditioned media. One hundred microlitres of conditioned media was added to 10 M Mca-KPLGL-Dpa-AR-NH2 -a broad spectrum MMP fluorogenic peptide substrate and incubated for 2 h at 37°C (R&D Systems Inc., Minneapolis, MN). As a control, conditioned media from untreated MCF-7 cells was spiked with IGF-1, IGF-1+MAPK, or IGF-1+PI3K inhibitors. Enzymatic activity was measured using a SpectraMax M2 microplate reader (Molecular Devices) and SoftMax Pro v5 software with excitation at 320 nm and emission at 405 nm.

### RNA Isolation

48 h after treatment, total RNA from MCF-7 cells was extracted using an RNAeasy mini kit (Qiagen) according to the manufacturer's instructions. The integrity and purity of the total RNA were checked using gel electrophoresis and a NanoVue Spectrophotometer (GE Healthcare).

### Western Blot Analysis

MCF-7 cells were treated for 1 h with MAPK or PI3K inhibitors and then protein was isolated from cells using M-PER Mammalian Protein Extraction Buffer (Thermo Scientific). Samples were resolved using a 10% polyacrylamide gel and transferred to a Millipore Immobilon-FL polyvinylidene difluoride membranes (Millipore). Membranes were then blocked in Odyssey Blocking Buffer (OBB, LI-COR Biosciences) for 1 h at RT. Samples were then incubated for 16 h at 4°C with appropriate antibodies: anti-ERK (1:500 Cell Signaling), anti-phospho ERK (1:500 Cell Signaling), anti-AKT (1:500 Cell Signaling), anti-phospho AKT (1:500 Cell Signaling) and GAPDH (1:20000 Cell Signaling). Membranes were washed and incubated wit appropriate secondary antibody for 1 h at room temperature. Bands of immunoreactivity were visualized and images captured using an Odyssey 2.1 scanner (LI-COR Biosciences).

### Synthesis of Complementary DNA and Quantitative (real-time) PCR

Complementary DNA (cDNA) was synthesized using qScript™ cDNA SuperMix (Quanta BioSciences). Q-PCR was performed using the CFX96™ Real-Time PCR Detection System (Bio-Rad) in a two-step procedure using PerfeCTa¨ SYBR¨ Green SuperMix (Quanta Biosciences). Amplification of glyceraldehydes-3-phosphate dehydrogenase (GAPDH) was performed to standardize the amount of sample cDNA. Primer sequences were as follows: GAPDH (forward) 5'tcggtgtgaacggatttg, (reverse) 5'ggtctcgctcctggaaga;

E-Cadherin (forward) 5'gaccggtgcaatcttcaaaa, (reverse) 5'caggtctcctcttggctctg;

N-Cadherin (forward) 5'agcttctcacggccatacacc, (reverse) 5'gtgcatgaaggacagcctct;

Vimentin (forward) 5'ctggatttcctcttcgtgga, (reverse) 5'cgaaaacaccctgcaatctt;

Occludin (forward) 5'atgccatgggactgtcaact, (reverse) 5'tttgtgggacaaggacaca.

All reactions were performed in a 96 well plate using the following cycling conditions: 40 cycles of 95°C for 15s, and 60°C for 30 s, 72°C 1 min. Using the CT (delta-delta CT) method, the value of each control sample was set at 1 and used to calculate the fold-change of target genes. When comparing gene expression profiles between vehicle control and latent TGF-β treatment, we found no significant changes associated with EMT; accordingly, we normalized all other treatment conditions to latent TGF-β treatment in order to highlight the importance of TGF-β activation.

### Immunofluorescence

All cells were grown on glass coverslips pretreated with 1 mg/ml Poly-Lysine. After treatment cells were fixed with 4% paraformaldehyde for 10 mins and then rinsed three times with phosphate-buffered saline (PBS) at room temperature. For morphological analysis, cells were permeablized with 0.1% Triton-X 100 and stained with Alexa Fluor 633 phalloidin (1:33 dilution) for 20 mins and 4',6-diamidino-2-phenylindole (DAPI) for 5 mins. For β-catenin localization, after blocking with 10% normal goat serum (Gibco) for 1 h at room temperature, cells were incubated with rabbit polyclonal β-catenin antibody (1:100 dilution; Sigma-Aldrich) at room temperature for 2 h, washed three times with PBS, and incubated with Alexa Fluor 488 labelled goat anti-rabbit secondary antibody (1:200 dilution; Molecular Probes) for 1 h. The cells were then washed with PBS and counterstained with 4',6-diamidino-2-phenylindole (DAPI) for 10 mins. Cells were all mouted using ProLong¨ Gold antifade reagent (Invitrogen). Images were taken using a Zeiss Z1 Microscope, AxioVision MRm camera and AxioVision 4.8 software. Lenses used were a Zeiss 63× oil immersion 1.4NA and a Zeiss 20× 0.5NA. Images were taken at 23°C at the Integrated Microscopy @ Biotron - University of Western Ontario.

### Matrigel Invasion Assay

Eight hours after treatments, one hundred thousand cells were added per transwell invasion chamber coated with 1-2 mg/ml Matrigel (reconstituted basement membrane; BD Biosciences, Mississauga, ON). Cells were allowed to invade for 24 h. Cells were fixed for 30 mins in methanol, stained for 30 mins with 1% crystal violet and the number of invaded cells were counted per field of view at 20× magnification.

### Statistical Analysis

Statistical significance was determined using Tukey's Post Hoc Test based on 3 independent experiments.

## Abbreviations

DAPI: 4',6-diamidino-2-phenylindole; DMEM: Dulbecco's modified eagle's medium; ECM: extracellular matrix; EMT: epithelial to mesenchymal transisition; IGF: insulin: like growth factor; IGFBP: IGF-binding proteins; IGFR: insulin-like growth factor receptors; LAP: latency-associated peptide; LTBP: latent TGF-β binding protein; MAPK: mitogen activated protein kinase; MMP: matrix metalloproteinase; PBS: phosphate-buffered saline (PBS); PI3K: phosphoinositide 3-kinases; RTK: receptor tyrosine kinases; TGF-β: transforming growth factor beta

## Competing interests

The authors declare that they have no competing interests.

## Authors' contributions

LW participated in the design of the study, the writing and editing of the manuscript and carried out all experiments. SD participated in its design and coordination and helped to draft the manuscript. Both authors read and approved the final manuscript.
